# Circular RNA circPRMT5 is upregulated in breast cancer and is required for cell proliferation and migration

**DOI:** 10.3906/sag-2102-90

**Published:** 2021-09-29

**Authors:** Xiaofeng LI, Dairong ZHANG, Zuxi FENG, Xiangjing XU, Jihong ZHANG, Aiping YU, Li ZHU, Jie XIAO, Junhua DU, Min CHEN

**Affiliations:** 1 Department of Public Health Management, Faculty of Medicine, Second People’s Hospital of China Three Gorges University, Yichang, China; 2 Department of Urology Surgery, Faculty of Medicine, Second People’s Hospital of China Three Gorges University, Yichang, China; 3 Administrative Office, Second People’s Hospital of China Three Gorges University, Yichang Second People’s Hospital, Yichang, China; 4 Department of Nursing, Faculty of Medicine, Second People’s Hospital of China Three Gorges University, Yichang, China; 5 Department of Integrated Traditional and Western Medicine, Faculty of Medicine, Second People’s Hospital of China Three Gorges University, Yichang, China; 6 Department of Thyroid and Breast Tumor, Faculty of Medicine, Second People’s Hospital of China Three Gorges University, Yichang, China; 7 Department of Surgery, Faculty of Medicine, Second People’s Hospital of China Three Gorges University, Yichang, China; 8 Department of Medical Education, Faculty of Medicine, Second People’s Hospital of China Three Gorges University, Yichang, China; 9 Department of Tumor Chemoradiotherapy, Faculty of Medicine, Second People’s Hospital of China Three Gorges University, Yichang, China; 10 Department of Nosocomial Infection, Faculty of Medicine, Second People’s Hospital of China Three Gorges University, Yichang, China

**Keywords:** Breast cancer, circPRMT5, diagnosis, prognosis, proliferation, metastasis

## Abstract

**Background/aim:**

To evaluate the role of cyclic protein arginine methyltransferase 5 (circPRMT5) in the occurrence and development of breast cancer (BC).

**Materials and methods:**

A total of 90 BC patients who underwent radical mastectomy and 40 age-matched healthy female controls were recruited in the Second People’s Hospital of China Three Gorges University, Yichang Second People’s Hospital from 2017 to 2020. Quantitative reverse transcription polymerase chain reaction (RT-qPCR) was used to detect the expression levels of circPRMT5 in BC tissues, serum, normal breast cell line (MCF-10A), and BC cell line (T47D, MCF-7, BT549, Hs-578T, and MDA-MB-231, MDA-MB-468). The associations between circPRMT5 expression level and age, tumor size, degree of differentiation, TNM stage, distant metastasis, estrogen receptor (ER) or progesterone receptor (PR), and human epidermal growth factor receptor 2 (HER2) status were analyzed. BC cell lines with circPRMT5 knockdown or overexpression were subject to CCK-8 cell proliferation assay, and transwell cell invasion/migration assay.

**Results:**

CircPRMT5 expression in BC tissue was higher than that in adjacent normal breast tissue. Consistently, the expression level of circPRMT5 was also elevated in serum samples collected from BC patients when compared with healthy controls. And in multiple breast cancer cell lines, circPRMT5 was upregulated as compared to normal breast epithelial MCF-10A cells. CircPRMT5 expression level was correlated with tumor size, TNM stage, lymph node metastasis distant metastasis, but no correlation was observed with ER, PR, HER2 status. Overexpression of circPRMT5 promoted the proliferation, invasion, and migration of MCF7 cells; while the knockdown of circPRMT5 inhibited cell proliferation, invasion, and migration.

**Conclusion:**

CircPRMT5 seems to act as an oncogene in the progression of BC. Our data suggest that CircPRMT5 may be used as a biomarker for the diagnosis, prognosis evaluation, and targeted therapy of breast cancer.

## 1. Introduction

Breast cancer (BC) is one of the malignant tumors with the highest incidence in women [1]. The annual incidence and death toll account for 23% and 14% of the global diagnosis and deaths respectively, ranking as one of the leading causes of cancer-related fatality in females [2]. At present, there is no good biomarker for early diagnosis and prognostic evaluation of BC. Uncontrolled proliferation, invasion, and metastasis are important biological characteristics of BC cells, accounting for the recurrence and poor prognosis after initial treatment [3]. Distant metastasis has been considered one of the main causes of BC-related death [4]. Bone metastasis occurs in 70% of BC cases, which often correlates with a poor prognosis [5]. Therefore, the investigation of the molecular mechanism of BC growth and metastasis will provide new insight into novel biomarkers and the development of targeted therapy for effective BC diagnosis and treatment.

Circular RNA (circRNA) is a class of noncoding RNA molecule with a closed loop structure. The mechanism of circRNA molecule formation mainly involves “exon skipping” and “reverse shearing” of precursor mRNA [6]. Early studies proposed that circRNA molecules are redundant and nonfunctional molecules [7]. In recent years, the advancement of the deep sequencing technology enables the identification of a large number of exon or intron of circRNA, indicating that circRNAs are functional RNA molecules with regulatory properties [8,9]. Furthermore, the unique stable closed-loop structure of circRNA makes it a promising marker for clinical diagnosis. Indeed, several studies have revealed that circRNA molecules can be detected in the plasma of cancer patients, which are closely related to the survival of the patients [10–12].

CircPRMT5 (hsa_circ_0031242) is a circRNA implicated in the occurrence and development of a variety of malignant tumors [13, 14]. CircPRMT5 has been found to be highly expressed in hepatocellular carcinoma, bladder cancer, and nonsmall cell lung cancer, and it acts as an oncogene [15,16]. However, the role and the regulatory mechanism of circPRMT5 in BC remain to be uncovered. In this study, we collected BC tumor samples and adjacent normal tissues, as well as the serum samples from BC patients and healthy controls. We evaluated the expression level of circPRMT5 in BC tumor tissues and the serum sample of BC patients. We further investigated the functional role of circPRMT5 overexpression in BC cells.

## 2. Materials and methods

### 2.1. Patients and tissue samples

This study was approved by the Institutional Review Board of Second People’s Hospital of China Three Gorges University, Yichang Second People’s Hospital (LL-2016-011). Patient samples were obtained after written informed consent had been obtained, and all procedures were performed in this study involving human participants were in accordance with the Declaration of Helsinki (as revised in 2013). We collected BC tumor tissues and normal tissue adjacent to the tumor (paracancer tissues) from 90 patients during surgical resection at the Second People’s Hospital of China Three Gorges University, Yichang Second People’s Hospital between 2017 and 2020 (age range 18–80 years). Specimens were immediately frozen in liquid nitrogen after resection and stored at −80 °C until usage. Each sample (5 g) was used for total RNA extraction and the expression of circPRMT5 in these samples was compared by RT-qPCR.

**Figure 1:**
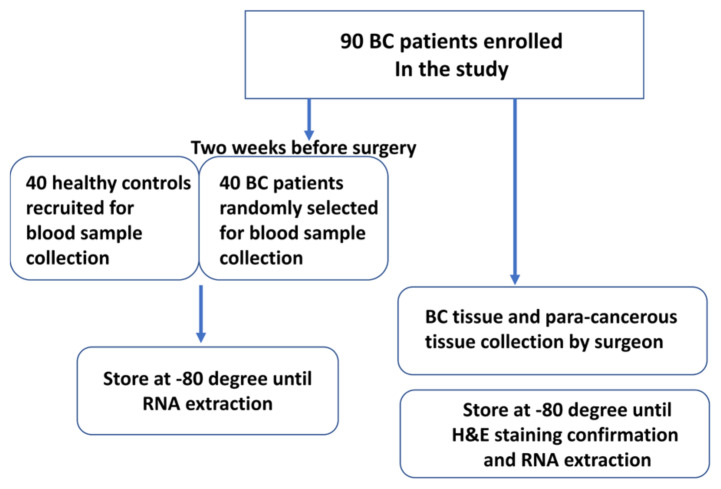
Flow diagram showing patient enrollment and clinical sample collection.

Inclusion criteria: Subjects who were able to provide written informed consent to participate in the study on a voluntary basis; subjects had no other concurrent malignant tumors; subjects with primary tumors that could be surgically removed, no blood-related diseases and infectious diseases were observed. Exclusion criteria: Subjects who were undergoing chemotherapy; subjects had fatal diseases; subjects with benign tumors, and patients with nonprimary breast lesions. All collected tumor tissues were diagnosed by three pathologists. The tumor site was confirmed by hematoxylin-eosin (H&E) staining, and then the tumor and paracancer tissues were separated. H&E staining was performed for a second time to further confirm the successful separation of tumor and nontumor tissues (see the flowchart in Figure 1). The pathological diagnosis of breast cancer was performed according to the WHO Classification ( https://radiopaedia.org/articles/who-classification-of-tumors-of-the-breast ) and the Elston and Ellis grading system ( http://tvmouse.ucdavis.edu/bcancercd/311/grading_diagram.html ).

Additionally, 2 weeks before the surgical operation, 40 out of 90 BC patients were randomly selected and the circPRMT5 expression level in serum was compared to that of 40 age-matched healthy female volunteers enrolled in the same hospital (see the flowchart in Figure 1). Peripheral venous blood samples were collected 2 weeks before the surgical operation in the BC patients to detect the expression of circPRMT5 in serum. All the 90 BC patients were followed up regularly by telephone every month after surgery, and the recurrence and survival status of patients were recorded. Relapse-free survival time was defined as the time interval from the date of surgery to the date of the first clinical diagnosis of recurrence, and censored data was defined as the time interval from the date of surgery to the date of the last follow-up of the patients.

### 2.2. Cell culture and transfection

Human BC cell lines (T47D, MCF-7, BT549, Hs-578T and MDA-MB-231, MDA-MB-468) and normal breast epithelial MCF-10A cells were obtained from the Cell Bank of Chinese Academy of Sciences (Shanghai, China). Cells were cultured in RPMI-1640 medium (Gibco, USA) supplemented with 10% fetal bovine serum and 1% penicillin/streptomycin at 37 °C in a humidified cell incubator with 5% CO_2_. Cell transfection procedure was performed as described below:

MCF7 cells were trypsinized and centrifuged at 500 × g for 5 min, and then resuspended in complete medium. Cells were seeded into a 6-well plate at a density of 5 × 10^5^ cells/well and incubated overnight. When the cell density reached 60% to 80% confluency, transfection of siRNA (small interfering RNA) was conducted using Lipofectamine™ RNAiMAX transfection reagent (Invitrogen, USA) according to the manufacturer’s manual. Briefly, 4 μL of nonspecific siRNA (si-NC) or siRNA targeting circPRMT5 (si-circPRMT5) (100 nM) were diluted with 200 μL of opti-MEM medium (Thermo Fisher Scientific, USA), respectively. Then 8 μL of RNAiMAX transfection reagent was mixed with 200 μL of opti-MEM medium. The diluted siRNA was mixed with transfection reagent at room temperature for 15 min, and then added to MCF7 cells. After 6 h, cells were replenished with fresh medium for 36 h incubation before subsequent experiments. siRNA targeting circPRMT5 and siRNA control were purchased from Genscript Biotech (Nanjing, China). The oligonucleotide sequences are as follows: si-circPRMT5 sense chain: 5′-CUCCUGACCUCAGUUCAUCTT-3′, antisense strand: 5′-GAUGAACUGAGGUCAGGAGTT-3′.

### 2.3. Overexpression of circPRMT5

The control vector and circPRMT5 overexpression plasmid pCDNA3.1-circPRMT5 were purchased from Genscript Biotech (Nanjing, China). The day before transfection, MCF7 cells were seeded in a 6-well plate at a density of 6 × 10^5^ cells per well to ensure that the cells reached 70% confluence before transfection. Lipofectamine 2000 reagent (5 μL) (Thermo Fisher Scientific, USA) was diluted with 125 μL of opti-MEM medium. Plasmid (2.5 μg) was diluted with 125 μL of opti-MEM medium. The diluted plasmid was mixed with Lipofectamine 2000 reagent (1:1) and incubated for 15 min at room temperature. The mixed plasmid liposome complex was added to the cell culture. After 6 h, fresh medium was replaced and cells were further cultured for 36 h before following experiments.

### 2.4. RNA isolation and RT-qPCR

Total RNA was isolated from serum, tissue, and BC cell lines using a Trizol reagent (Takara Biotechnology Co., Ltd., Dalian, China) according to the manufacturer’s instructions. The purity of the extracted RNA was determined by spectrophotometry at 260/280 nm, and 5μg purified total RNA was reverse transcribed into cDNA using the PrimeScript RT reagent kit (Cat# RR037A, Takara, China). Quantitative real-time PCR (qPCR) reactions were performed on ABI Prism 7500 real time PCR instrument (Applied Biosystems, USA) using the SYBR Green PCR Master Mix (Takara, Japan). The PCR conditions were as follows: one cycle of 50°C for 2 min and 95°C for 10 min, followed by 40 cycles of 95 °C for 30 s and 60 °C for 30 s. The relative expression of the target gene was determined by 2–ΔΔCt method. GAPDH was selected as the internal control gene for normalizing the expression. The primer sequences used for RT-qPCR were listed below [16]: GAPDH forward primer 5′- TCCCATCACCATCTTCCAGG -3′, reverse primer 5′-GATGACCCTTTTGGCTCCC-3′; circPRMT5 forward primer 5′- TACCATTGGCCTCTAGCCCT-3′, reverse primer 5′- CAAGGGGAATCACAGCCCAT-3′.

### 2.5. CCK-8 (cell counting kit-8) assay for cell proliferation

CCK-8 cell proliferation assay was performed according to a previous study [17]. Cell counting kit-8 (CCK-8; Dojindo, Japan) assay was used for the cell proliferation capacity. Cells were seeded in a 96-well plate at a density of 3000 cells/well, and cultured for indicated time period. CCK-8 solution (10 μL) was added to the cell culture at each time point, the plates were incubated for 3 h. CCK-8 (10 μL) reagent was added to each well at the indicated time point and incubated in the incubator for 1 h. A microplate reader (Bio-Rad, CA, USA) was used to detect the absorbance value (OD value) in each well at 450 nm.

### 2.6. Cell migration and invasion assays

Cell migration and invasion assays were performed according to a previous study [18]. Corning Transwell polycarbonate membrane cell culture inserts with 8.0-μm pores were uncoated for migration assay, or coated with 50 μL of BD Matrigel Basement Membrane Matrix (BD Biosciences, USA) diluted 1:3 with FBS-free DMEM (fetal bovine serum free Dulbecco’s Modified Eagle Medium) for invasion assay. Cells were trypsinized and then resuspended in serum-free medium. 2 × 10^4^ cells were seeded in the top chamber with serum-free medium and allowed to migrate toward serum-containing medium in the lower chamber for 24 h. Then cells were fixed in 4% paraformaldehyde and stained with 0.01% crystal violet (AS1086, ASPEN, China) for 10 min. After wiping the cells remaining in the upper chamber with cotton swabs, the migratory or invasive cells were imaged and counted using a Leica AM6000 microscope (Leica Microsystems, Germany). The numbers of migratory or invasive cells were calculated as the average numbers of five random fields per well under 100× magnification.

### 2.7. Statistical analysis

Statistical analyses were performed with SPSS 20.0 software (IBM SPSS, Armonk, NY, USA). All the experiments were repeated three times. CircPRMT5 expression in tissues was examined in 90 pairs of cancer and paracancerous normal tissues; circPRMT5 expression in serum was analyzed from the blood samples of 40 BC patients and 40 healthy controls. The association between circPRMT5 expression in cancer tissues and the clinic pathological parameters was evaluated with chi-square analysis. The statistical difference between the two groups was compared using unpaired student’s t-tests. Comparisons among multiple groups were analyzed using one-way analysis of variance (ANOVA) with Tukey’s post hoc test for pairwise comparison. Comparisons of data at multiple time points were examined using two-way ANOVA. Kaplan Meier Curve and log-rank test were used to compare the cumulative survival rates in 90 BC patients. Data were reported as the mean ± standard deviation (SD). P < 0.05 was considered to be statistically different.

## 3. Results

### 3.1. Expression of circPRMT5 in BC

**Figure 2:**
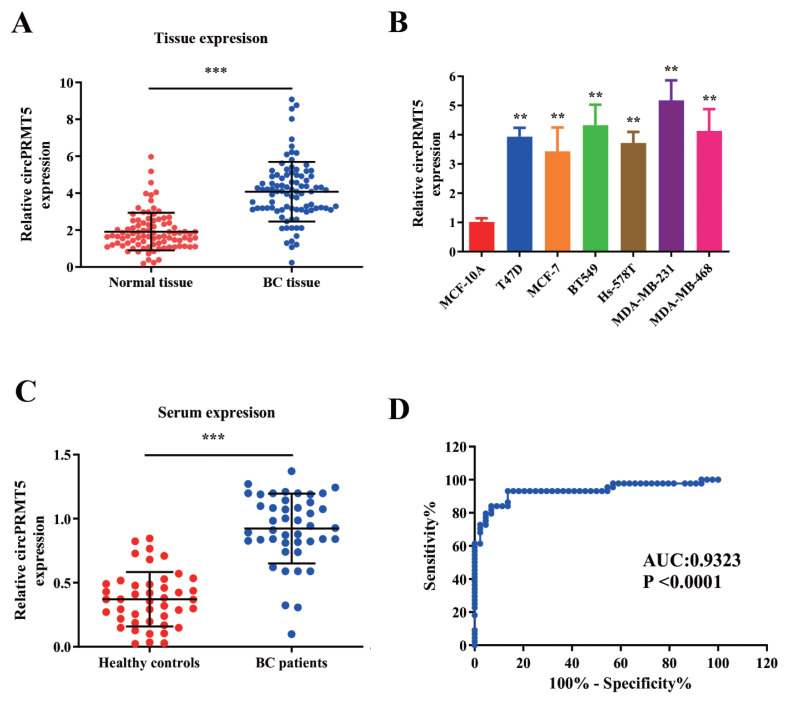
Expression of circPRMT5 in BC. A: the RT-qPCR method detects the expression level of circPRMT5 in 90 pairs of BC tumor tissues and adjacent normal tissues (normal, paracancer tissues). CircPRMT5 expression was significantly increased in BC tumor samples, p < 0.001. B: RT-qPCR method was used to measure the expression level of circPRMT5 in BC cell lines (T47D, MCF-7, BT549, Hs-578T, MDA-MB-231, and MDA-MB-468) and normal breast epithelial MCF-10A cells. The expression of circPRMT5 in BC cell lines were significantly elevated, p < 0.01. C: RT-qPCR method was used to detect the expression level of circPRMT5 in the serum samples of 40 cases of BC patients and 40 normal healthy controls. CircPRMT5 expression in BC serum was significantly increased, p < 0.001. D: Receiver operating characteristics (ROC) curve of circPRMT5 expression level for predicting the BC cancer from the 90 BC tumor samples and 90 paracancerous tissues. AUC (area under the curve) >0.8 indicates a good prediction with p < 0.001.

To evaluate circPRMT5 expression in BC tumor samples, we used RT-qPCR to compare the expression level of circPRMT5 in 90 pairs of BC tissues and paired paracancerous tissues. The results showed that circPRMT5 expression in BC tumors was significantly higher than that of paracancerous tissues (P < 0.001, Figure 2A). Consistently, circPRMT5 expression level in multiple BC cell lines was also significantly increased than that in normal cells (P < 0.01, Figure 2B). In addition, the levels of circPRMT5 collected from serum in BC patients were also elevated when compared with healthy controls (P < 0.001, Figure 2C). Moreover, the receiver operating characteristics (ROC) curve of circPRMT5 expression level was used for predicting the BC cancer status from 90 BC tumor samples and 90 paracancerous tissues. AUC (area under the curve) >0.8 indicates that circPRMT5 expression with a cut-off value of 3.0 could be a good predictor for breast cancer with a 78.6% sensitivity and 91.3% specificity (Figure 2D). As shown in Table, 90 patients were divided into low and high expression groups based on the median value of circPRMT5 expression, and a high circPRMT5 expression level was associated with tumor maximum diameter, degree of differentiation, TNM staging, lymph node metastasis distant metastasis (P < 0.05). However, circPRMT5 expression level was not associated with ER, PR, HER2 status (P > 0.05) (Table).

### 3.2. Relationship between circPRMT5 and prognosis of BC

**Figure 3:**
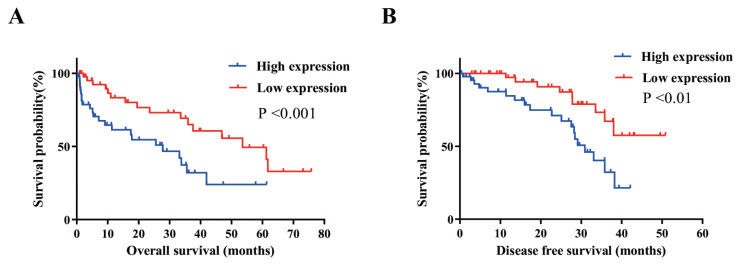
Relationship between circPRMT5 and prognosis of BC patients. A: Kaplan–Meier survival curve was used to assess the overall survival of BC patients in circPRMT5 low expression group (n = 45) and circPRMT5 high expression group (n = 45). 90 patients were divided into low and high expression group based on the median value of circPRMT5 expression detect by qPCR in Figure 1A. BC patients in the circPRMT5 high expression group showed a poorer survival than that of low expression group, p < 0.001. B: Kaplan–Meier survival curve was used to evaluate the disease-free survival time of BC patients in circPRMT5 low expression group (n = 45) and the circPRMT5 high expression group (n = 45). BC patients in the circPRMT5 high expression group had worse progression-free survival than that of low expression group, p < 0.01.

To examine whether circPRMT5 expression level is correlated with the prognosis of BC patients, we divided the 90 patients into low expression group (n = 45) and high expression group (n = 45) based on the median value of circPRMT5 expression detected by qPCR in Figure 1A. From the Kaplan–Meier survival curve, the overall survival time and progression-free survival time of BC patients in the circPRMT5 high expression group were significantly shorter than that of the low expression group (P < 0.001) (Figure 3). Taken together, the data suggest that circPRMT5 is upregulated in BC and higher circPRMT5 expression correlates with a poor prognosis in BC patients.

### 3.3. Functional analysis of circPRMT5 overexpression and silencing on proliferation, invasion, and migration in BC cell line

**Figure 4:**
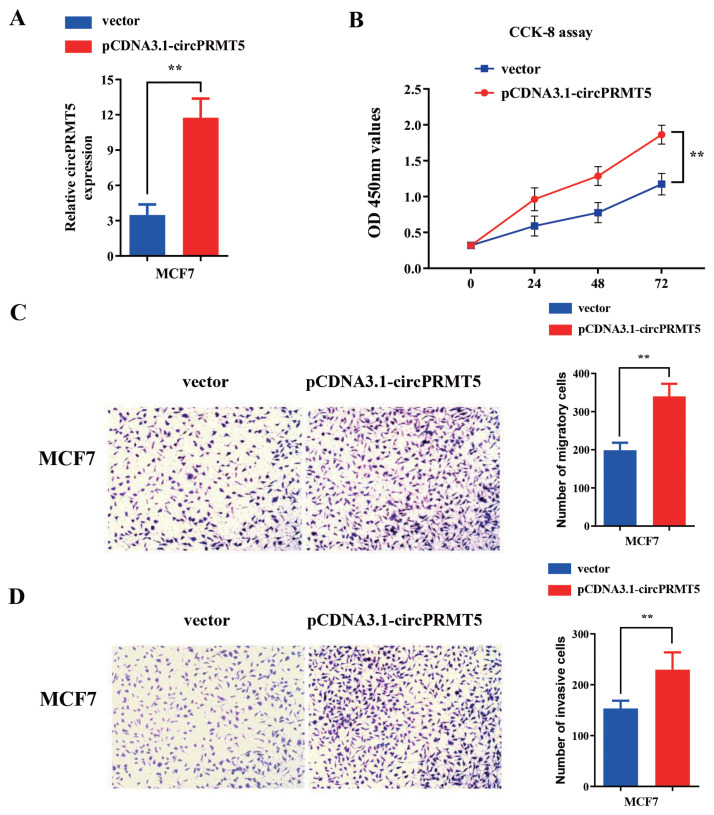
Functional assay of circPRMT5 overexpression on proliferation, invasion, and migration in MCF-7 cell line. A: RT-qPCR method was conducted to detect the overexpression efficiency compared with vector. A: transfection with pcDNA3.1-circPRMT5 can effectively overexpress circPRMT5 in cells, p < 0.01. B: CCK-8 method detects the light absorption value of 0h, 24h, 48h, and 72h at 450nm wavelength in different groups (vector and pCDNA3.1-circPRMT5) in MCF7 cells. C: Transwell chamber (without matrigel) experiment was used to test the migration ability in different groups (vector and pCDNA3.1-circPRMT5) of MCF7 cells. D: Transwell chamber (adding Matrigel) experiment to detect the invasion ability in different groups (vector and pCDNA3.1-circPRMT5) of MCF7 cells.

From the results in Figure 1B, MCF7 cells showed a relatively lower expression level of circPRMT5 than other BC cell lines. We selected MCF7 cell line for overexpression experiment using plasmid pCDNA3.1-circPRMT5. The functional effects of circPRMT5 in the proliferation, invasion, and migration were analyzed. RT-qPCR analysis showed that transfection of pCDNA3.1-circPRMT5 was capable to increase circPRMT5 expression by more than four times in MCF7 cells **(**Figure 4A). CCK8 proliferation assay showed that the overexpression of circPRMT5 could promote cell proliferation (Figure 4B). Transwell migration and invasion assays demonstrated that circPRMT5 overexpression enhanced the migration and invasion ability of MCF7 cells (Figure 4C and D). Collectively, these results indicated that elevated circPRMT5 expression promotes cell proliferation, migration, and invasion in BC cells.

**Figure 5:**
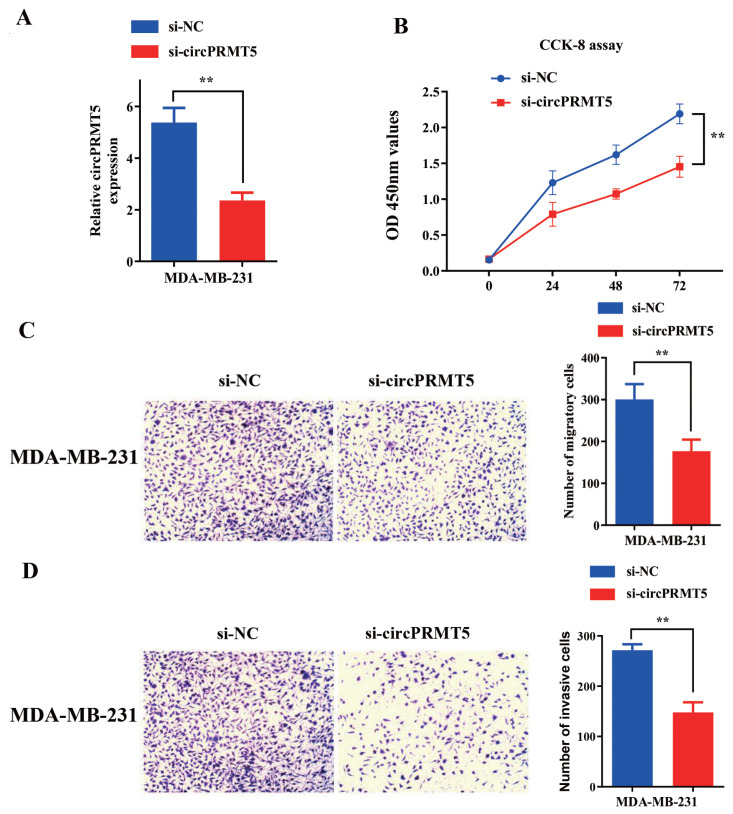
Functional assay of circPRMT5 overexpression on proliferation, invasion, and migration in MDA-MB-231cell line. A: Select the BC cell line with the highest circPRMT5 expression (MDA-MB-231) from the results in Figure 1B for knockdown using si-circPRMT5, and the RT-qPCR method detects knockdown efficiency. B: CCK-8 method detects the light absorption value of 0h, 24h, 48h, and 72h in different groups (si-NC and si-circPRMT5) at 450nm wavelength in MDA-MB-231 cells. C: Transwell chamber (without matrigel) experiment checks the migration ability of different groups (si-NC and si-circPRMT5) of MDA-MB-231 cells. D: Transwell chamber (adding Matrigel) experiment to detect the invasion ability of different groups (si-NC and si-circPRMT5) of MDA-MB-231 cells.

To further confirm an indispensable role of circPRMT5 in BC cells, we knocked down circPRMT5 using siRNA specifically targeting circPRMT5 in MDA-MB-231 cells (Figure 5A). In comparison with the control group, the proliferation of MDA-MB-231 cells with circPRMT5 knockdown was significantly suppressed (Figure 5B). Cell migration and invasion assay further showed that the number of migratory and invasive BC cells were largely reduced after circPRMT5 silencing (Figure 5 C and D). Taken together, these results suggest that circPRMT5 is indispensable for supporting cell proliferation, migration, and invasion in BC cells.

## 4. Discussion

In the study, we found that circPRMT5 was upregulated in BC tissue as compared to adjacent normal breast tissue. Consistently, circPRMT5 was also upregulated in serum samples collected from BC patients when compared with healthy controls. Importantly, a higher circPRMT5 expression level correlated with a poor prognosis in BC patients. The overexpression of circPRMT5 in BC cells promoted the proliferation, invasion, and migration; while the knockdown of circPRMT5 inhibited cell proliferation, invasion, and migration. These data suggest that circPRMT5 functions as an oncogenic factor in BC.

Many risk factors including age, hormone levels, fertility, breastfeeding, and gene mutations have been reported to be responsible for BC occurrence [19,20]. The metastasis of the Her2-negative invasive breast cancer cells is linked with poor prognosis [21, 22]. Previous studies have been revealed genetic mutations contributing to BC predisposition. Mutations or dysregulation of tumor-suppressor genes, such as BRCA-1, BRCA-2, p53, and PTEN play an important role in the initiation and development of BC [23–25]. Importantly, accumulating evidence uncovers the critical role of noncoding RNA such cicrRNAs in the progression of BC, which may regulate different biological processes by sponging microRNA. For example, the overexpression of circ-ABCB10 and circ_001783 were found to facilitate BC progression through sponging miR-1271 and miR-200c-3p, respectively [26, 27]. In addition, circ_0003645 was shown to promote BC proliferation by targeting miR-139-3p/HMGB1 signaling axis [28], and circ_0001667 could enhance BC cell survival by regulating TAZ in Hippo signal pathway [29]. In our study, we also demonstrated the upregulation of CircPRMT5 in BC tumors and cell lines, and the silencing of CircPRMT5 suppressed the aggressive phenotype of BC cells. We therefore propose that CircPRMT5 may function as an oncogene in BC development.

CircPRMT5 is one of the circRNAs whose abnormal expression has been documented in a variety of malignant tumor tissues. circPRMT5 is highly expressed in gastric cancer patients, and the upregulation of circPRMT5 can promote the expression of oncogene MYC through sponging miR-145 and miR-1304 [30]. In bladder cancer, overexpressed circPRMT5 regulates the epithelial-mesenchymal transition of bladder cancer cells and promotes the metastasis [13]. Moreover, the expression level of circPRMT was also elevated in esophageal cancer cells to augment the migration [31]. In hepatoma and nonsmall cell lung cancer (NSCLC), upregulation of circPRMT5 facilitates cancer progression via targeting HK2 or EZH2 [15,16]. Therefore, it seems that circPRMT5 plays multifaceted roles by targeting different pathways to support tumorigenesis in different cancers.

In line with these previous findings, our present study showed that the expression level of circPRMT5 is significantly increased in human BC tissues, serum samples of BC patients and BC cells. High expression of circPRMT5 is associated with a poorer prognosis in BC patients. These findings are consistent with a recent study in which the upregulation of circPRMT5 in breast cancer cells contributes to the aggressive phenotype by mediating the miR-509-3p/TCF7L2 axis to activate the PI3K/AKT pathway [32]. Apart from that, our study further demonstrated that circPRMT5 is indispensable for the invasion and migration capabilities of BC cells. Furthermore, the clinical parameter analysis showed that a high circPRMT5 expression level is correlated with larger tumor size, more advanced TNM stage, poorer differentiation, and more lymph node metastasis distant metastasis. Together, our results and the recent study highlighted a functional role of circPRMT5 in supporting the malignant phenotype in BC cells. However, but no correlation was observed between circPRMT5 and ER, PR, HER2 status, indicating that the upregulation of circPRMT5 in BC may not result from dysregulated hormone receptor signaling.

To sum up, our study found that circPRMT5 is highly expressed in BC tumor and the plasma of BC patients. The higher circPRMT5 expression level is related to more aggressive tumor phenotypes including more advanced tumor staging, lymph node metastasis, and poorer differentiation in tumor. Our data suggest that circPRMT5 might be a potential diagnostic and prognostic biomarker for BC. However, this study is limited to the analysis of clinical samples and the functional assays in cell experiments. Further experiments are needed to validate the tumorigenic role of circPRMT5 in BC animal model and to elucidate the molecular targets of circPRMT5.

## Figures and Tables

**Table:** Correlations of CircPRMT5 expression with clinicopathologic features of breast cancer.

Parameters		Total	CircPRMT5		P-value
			Low/high		
Age	>50	38	15	23	0.088
	≤50	52	30	22	
Tumor size	>2cm	34	25	9	0.001
	≤ 2cm	56	20	36	
Differentiation	High (well)	36	28	8	0.001
	Low (poor)	54	17	37	
TNM stage	I—II	39	28	11	0.001
	III—IV	51	17	34	
Lymph node metastasis	Negative	43	31	12	0.001
	Positive	47	14	33	
Distant metastasis	M0	58	39	19	0.001
	M1	32	6	26	
ER status	Positive	28	15	13	0.649
	Negative	62	30	32	
PR status	Positive	26	9	17	0.063
	Negative	64	36	28	
HER-2 status	Positive	26	11	15	0.352
	Negative	64	34	30	
